# Cardiovascular adverse effects of common non-oncologic medications: from mechanisms to clinical management

**DOI:** 10.1093/ehjcvp/pvag007

**Published:** 2026-02-02

**Authors:** M Cecilia Bahit, Pilar Agudo-Quilez, Jose Zamorano, C Michael Gibson

**Affiliations:** Baim Institute for Clinical Research, 930 Commonwealth Ave, Boston, MA 02215, USA; INECO Neurociencias, Rosario, Santa Fe, 2000, Argentina; Cardiology Department, Hospital Universitario Ramón y Cajal, Madrid 28034, Espana; Cardiology Department, Hospital Universitario Ramón y Cajal, Madrid 28034, Espana; Baim Institute for Clinical Research, 930 Commonwealth Ave, Boston, MA 02215, USA; Division of Cardiovascular Medicine, Department of Medicine, Beth Israel Deaconess Medical Center, Harvard Medical School, Boston, MA 02215, USA

**Keywords:** Cardiovascular adverse effects, Cardiotoxicity, Cardiac safety, Non-oncologic drugs

## Abstract

Cardiovascular adverse effects of drugs have significant practical implications for patient management. While cardiovascular adverse effects have commonly been associated with oncologic therapeutics, a growing body of evidence suggests that non-oncologic medications can also be associated with significant cardiovascular harm. These adverse effects range from arrhythmias, conduction abnormalities, QT prolongation, heart failure, myocardial infarction, or structural cardiomyopathy. Non-oncologic drugs that have been implicated include antibiotics (e.g. macrolides, fluoroquinolones), antidiabetics (e.g. thiazolidinediones), non-steroidal anti-inflammatory drugs, drugs for gastrointestinal and urological conditions, and most importantly, cardiovascular drugs. In this narrative review, we focus on the most common non-oncologic drugs that cause cardiovascular adverse effects, their proposed underlying mechanisms with particular emphasis on their clinical manifestations and clinical implications for everyday cardiovascular practice.

## Graphical Abstract

**Graphical Abstract:**
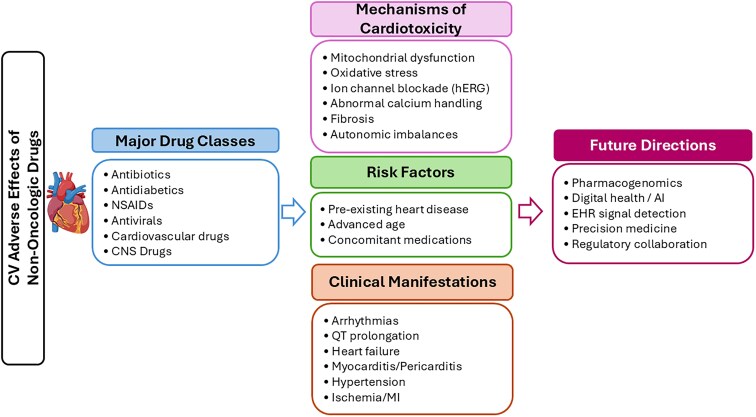
**CV Adverse Effects of Non-Oncologic Drugs.** This graphical abstract demonstrates cardiovascular adverse effects of non-oncologic drugs and is described by the major drug classes, mechanisms of cardiotoxicity, risk factors, clinical manifestations, and future directions. AI, artificial intelligence; CNS, central nervous system; CV, cardiovascular; EHR, electronic health record; hERG, human ether-à-go-go-related gene; MI, myocardial infarction; NSAIDS, non-steroidal anti-inflammatory drugs

## Introduction

Cardiovascular adverse effects are a common cause of emergency department visits and hospitalizations. In older individuals with prior cardiovascular disease, adverse cardiovascular effects are the most frequent cause of hospital admissions.^1,2^ While cardiovascular adverse effects are most commonly associated with oncologic medications, a growing body of evidence suggests that commonly used non-oncologic medications can also be associated with significant cardiovascular harm.^3,4^ These effects may range from arrhythmias, conduction abnormalities, QT prolongation, heart failure (HF), myocardial infarction (MI), myocarditis, pericarditis, or structural cardiomyopathy. The underlying mechanisms include disruption of ionic processes, which leads to induction of damage to the cells either directly or through impaired mitochondrial function or hypercoagulabity.^3^ Several non-oncologic drugs that have been associated with adverse effects include antibiotics^4^ (e.g. macrolides, fluoroquinolones), antidiabetics (e.g. thiazolidinediones), non-steroidal anti-inflammatory drugs (NSAIDs),^5^ drugs for gastrointestinal and urological conditions, and most importantly, cardiovascular drugs themselves.^3^

Cardiac safety concerns often form the basis for stopping a clinical trial or withdrawal of an approved agent from the market.^6^ The broad spectrum of cardiovascular adverse effects make the issue more challenging, and regulatory agencies have called for finding more sensitive and efficient methods to assess such deleterious cardiovascular effects.^6^ Additionally, the extent or severity of effects may depend on duration of exposure, dose, and certain patient characteristics.^6–9^ Cardiovascular toxicity may be amplified by pre-existing heart disease, advanced age, and concurrent therapies. These conditions can disrupt endogenous cardioprotective mechanisms, increasing susceptibility to arrhythmias, contractile dysfunction, and cardiomyocyte death. Cardiovascular adverse effects may remain undetected during safety testing in healthy subjects and only become apparent in disease states—a phenomenon known as ‘hidden toxicity’.^10^

Here, we review the most common non-oncologic drugs that cause cardiovascular adverse effects, their proposed underlying mechanisms, and practical considerations for prevention.

## Methods

### Search strategy

A comprehensive literature search was conducted to identify studies on cardiovascular adverse effects of the selected non-oncologic drug classes (NSAIDs, antidiabetic agents, antibiotics, antihypertensives, and antiarrhythmics). We searched multiple databases, including PubMed/MEDLINE, Embase, Web of Science, and the Cochrane Library, which focus on the last decade. The search was limited to English-language publications in peer-reviewed journals. We used combinations of keywords and MeSH terms for each drug class (e.g. ‘non-steroidal anti-inflammatory drugs’) combined with terms for cardiovascular outcomes and toxicity (e.g. ‘heart failure’) and mechanistic keywords (e.g. ‘mitochondrial dysfunction’). We also manually screened reference lists of key articles to identify additional studies. The following therapeutic domains are included in the main manuscript: antimicrobials, immunosuppressants and anti-inflammatory drugs, antidiabetic medications, and certain cardiovascular drugs. Central nervous system drugs, gastrointestinal and genitourinary system agents, and antiallergic medications are included in *Table 1*.

**Table 1:** List of selected drugs and cardiotoxicity

Drug	Mechanism of action	Toxic effect on the heart	Mechanism of cardiac toxicity	Withdrawn
**Central nervous system agents**				
Haloperidol^11^ (typical antipsychotic drug*)*	DRD1/2 blocker, GRIN2B blocker, DRD3 inverse agonist	QT prolongation, TdP, sudden cardiac death	IKr, INa, ICaL inhibition	
Thioridazine^11^ (first generation antipsychotic drug)	Blocks ADRA1A/B, DRD1/2, HTR2A; inhibits KCNH2	QT prolongation, TdP, sudden cardiac death	INaL, IKr inhibition	
Sertindole^12^ (second generation antipsychotic drug)	DRD2, HTR2A/C, HTR6 blocker	QT prolongation, TdP, sudden cardiac death	IKr inhibition	Withdrawn from the market
Ziprasidone^11^ (atypical antipsychotic)	Broad blocker of dopamine, serotonin, adrenergic, cholinergic receptors; HTR1A agonist	QT prolongation, TdP, sudden cardiac death	IKr inhibition	
Clozapine^13,14^ (atypical antipsychotics)	Broad-spectrum receptor blockade (dopamine, serotonin, histamine, adrenergic, cholinergic)	Myocarditis, cardiomyopathy	Unknown	
Dexfenfluramine^15,16^ (nonamphetamine anorectics)	SLC6A4 inhibitor	Valvular heart disease	HTR2B-induced activation of TGF-β signalling	Withdrawn from the market
Ergotamine^17^ (alpha-1 selective adrenergic agonis)	ADRA1A, DRD2, HTR1B/1D/2A agonist	Valvular heart disease	Fibrosis via HTR2B-induced activation of TGF-β signalling	
Fenfluramine^16,18^ (MAOI Type B)	SLC6A4 inhibitor, HTR2B blocker	Valvular heart disease	HTR2B-induced activation of TGF-β signalling	Withdrawn from the market
Fluoxetine^19^ (SSRIs)	SLC6A4 inhibitor	Bradycardia	ICaL & IKr inhibition; hERG trafficking inhibition	
Venlafaxine^20^ (SNRIs)	SLC6A4/2 inhibitor	QT prolongation, arrhythmias	INa inhibition	
Sibutramine^21–23^ (SNRI)	Inhibits SLC6A4/2/3	Myocardial infarction	IKr inhibition	Withdrawn from the market
Propoxyphene^24^ (opioid)	OP1/2/3 agonist	QT prolongation, TdP	IKr inhibition	Withdrawn from the market
Levomethadyl Acetate^25^ (synthetic opioid)	OPRM1 agonist	QT prolongation, TdP	IKr inhibition	Withdrawn from the market
Methysergide^26^ (semisynthetic ergot derivative)	HTR2A/B/C and HTR7 blocker; HTR1A agonist; binds HTR1B/E/F	Valvular heart disease	Fibrosis via HTR2B-induced activation of TGF-β signalling	
Pergolide^26,27^ (ergot-derived dopamine receptor agonist)	Broad dopamine and serotonin receptor blocker (HTR2B included)	Valvular heart disease	Fibrosis via HTR2B-induced activation of TGF-β signalling	
**Antimicrobial agents**				
Azithromycin^28^ (macrolide)	Inhibits 23S rRNA, rpID, rpIV, PADI4	QT prolongation, TdP, cardiac death	Mitochondrial toxicity	
Clarithromycin^29,30^ (macrolide)	Inhibits rpIJ, SLCO1B1, SLCO1B3	QT prolongation, myocardial infarction, arrhythmias, cardiac death	Mitochondrial toxicity	
Erythromycin (macrolide)	Inhibits 23S rRNA, MLNR, KCNH2, ALB	QT prolongation, ventricular tachycardia, TdP, VF	Mitochondrial toxicity	
Grepafloxacin^31^ (fluoroquinolones)	Inhibits gyrA, parC	QT prolongation	IKr inhibition	
Sparfloxacin^31^ (fluoroquinolones)	Inhibits parC, gyrA, TOP2A	QT prolongation	IKr inhibition	
Sofosbuvir^32^ (antiviral)	Inhibits NS5B	Bradycardia	Unknown	
Azidothymidine^33^ (antiviral)	Inhibits Pol, TERT	Dilated cardiomyopathy	Mitochondrial toxicity	
Pentamidine^34–36^ (antifungal)	Inhibits RNA transfer	QT prolongation, arrhythmias	hERG trafficking inhibition	
**Anti-inflammatory agents**				
Diclofenac^37,38^	Inhibits COX-1/2, SCN4A, ASIC1; potentiates ALOX5	Myocardial infarction	Prostacyclin synthase blockade	
Etoricoxib^37,38^	COX-2 inhibition	Thrombotic events	Prostacyclin synthase blockade	Withdrawn from the market
Rofecoxib^39^	COX-2 inhibition	Myocardial infarction	Prostacyclin synthase blockade	Withdrawn from the market
Ibuprofen^37,38^	COX-1/2 inhibition	Myocardial infarction, hypertension	Prostacyclin synthase blockade	
Naproxen^37^	COX-1/2 inhibition	Myocardial infarction	Prostacyclin synthase blockade	
**Antidiabetic medications**				
Alogliptin^40^	DPP4 inhibitor	Heart failure	Unknown	2013–NA
Rosiglitazone^41^	PPARG activator	Heart failure	Unknown	1994–NA
Saxagliptin^42^	DPP4 inhibitor	Heart failure	Unknown	2009–NA
**Cardiovascular agents**				
Dofetilide^43^ (class III antiarrhythmic agent)	Inhibits KCNK2, KCNJ12	QT prolongation, TdP	IKr inhibition	Withdrawn from the market
Encainide^44^ (class Ic antiarrhythmic agent)	Inhibits SCN5A	QT prolongation, TdP	IKr inhibition	Withdrawn from the market
Amiodarone (class III antiarrhythmic agent)	Inhibits K+, Ca2+ and Na+	QT prolongation	block K+, Ca2+, and Na+	
**Gastrointestinal agents**				
Cisapride^45^	Blocks HTR2A, HTR3A, HTR4; inhibits KCNH2	Ventricular arrhythmia, QT prolongation, TdP, cardiac arrest	IKr inhibition	Withdrawn from the market
Loperamide^46,47^	Blocks OPRM1, OPROD1, OPRK; inhibits POMC; modulates CALM1	Cardiac arrest, QT prolongation, ventricular tachycardia, TdP	Inhibition of voltage-gated calcium channels	
Omeprazole^48^	Inhibits ATP4A	Acute myocardial infarction, heart failure	Disruption of NO synthesis via ADMA production	
Tegaserod	Blocks HTR2A, HTR2B, HTR2C, HTR4	Ischaemia	IKr inhibition	Withdrawn from the market
**Genitourinary system agent**				
Terodiline^49^	Blocks muscarinic acetylcholine receptors	Ventricular tachycardia, cardiac death	IKr inhibition, blocking of calcium cycling	Withdrawn from the market
**Antiallergic agents**				
Astemizole^50^	HRH1 blocker, KCNH2 inhibitor	Long QT syndrome, TdP	IKr inhibition	Withdrawn from the market
Terfenadine^51^	HRH1 blocker	QT prolongation, TdP	IKr inhibition	Withdrawn from the market
Diphenhydramine^52^	HRH1, CHRM2 Blocker	QT Prolongation	Ikr Inhibition	

23S rRNA, 23s ribosomal ribonucleic acid; ADMA, asymmetric dimethylarginine; ADRA1A, alpha-1A adrenergic receptor; ADRA1B, alpha-1B adrenergic receptor; ALB, albumin, ALOX5, arachidonate 5-lipoxygenase; ASIC1, acid-sensing ion channel 1; ATP4A, ATPase H+/K+ transporting alpha subunit; Ca2+, calcium ion; CALM, calmodulin 1; CHRM2, cholinergic receptor muscarinic 2; COX-1, cyclooxygenase-1 (constitutive enzyme); COX-2, cyclooxygenase-2 (inducible enzyme); DPP4, dipeptidyl peptidase-4; DRD1, dopamine receptor D1; DRD2, dopamine receptor D2; DRD3, dopamine receptor D3; GRIN2B, glutamate ionotropic receptor, NMDA type subunit 2B; gyrA, DNA gyrase subunit A; hERG, human ether-à-go-go-related gene; HRH1, histamine receptor H1; HTR1A, 5-hydroxytryptamine receptor 1A (serotonin receptor 1A); HTR1B, 5-hydroxytryptamine receptor 1B (serotonin receptor 1B); HTR1D, 5-hydroxytryptamine receptor 1D (serotonin receptor 1D); HTR1E, 5-hydroxytryptamine receptor 1E (serotonin receptor 1E); HTR1F, 5-hydroxytryptamine receptor 1F (serotonin receptor 1F); HTR2A, 5-hydroxytryptamine receptor 2A (serotonin receptor 2A); HTR2B, 5-hydroxytryptamine receptor 2B (serotonin receptor 2B); HTR2C, 5-hydroxytryptamine receptor 2C (serotonin receptor 2C); HTR3A, 5-hydroxytryptamine receptor 3A (serotonin receptor 3A); HTR4, 5-hydroxytryptamine receptor 4 (serotonin receptor 4); HTR6, 5-hydroxytryptamine receptor 6 (serotonin receptor 6); HTR7, 5-hydroxytryptamine receptor 7 (serotonin receptor 7); ICaL, L-type calcium current; IKr, rapid delayed rectifier potassium current; Ina, fast sodium current; INaL, late sodium current; K+, potassium ion; KCNH2, potassium voltage-gated channel subfamily H member 2; KCNJ12, potassium inwardly rectifying channel subfamily J member 12; MAOI, monoamine oxidase inhibitor; Na+, sodium ion; NO, nitric oxide; NS5B, non-structural protein 5B (RNA-dependent RNA polymerase in HCV); OP1, opioid receptors 1 (delta); OP2, opioid receptors 2 (kappa); OP3, opioid receptors 3 (mu); OPRK, opioid receptor kappa type; OPRM1, opioid receptor mu 1; OPROD1, opioid receptor delta 1; PADI4, peptidyl arginine deiminase 4; parC, DNA topoisomerase IV subunit C; Pol, polymerase; POMC, pro-opiomelanocortin; PPARG, peroxisome proliferator-activated receptor gamma; RNA, ribonucleic acid; rpID, ribosomal protein L1; rpIJ, ribosomal protein L10; rpIV, ribosomal protein L23; SCN4A, sodium voltage-gated channel alpha subunit 4; SCN5A, sodium voltage-gated channel alpha subunit 5; SLC6A2, solute carrier family 6 member 2 (norepinephrine transporter—NET); SLC6A3, solute carrier family 6 member 3 (dopamine transporter—DAT); SLC6A4, solute carrier family 6 member 4 (serotonin transporter—SERT); SLCO1B1, solute carrier organic anion transporter family member 1B1; SLCO1B3, solute carrier organic anion transporter family member 1B3; SNRI, serotonin-norepinephrine reuptake inhibitor; SSRI, selective serotonin reuptake inhibitor; TdP, torsades de pointes; TERT, telomerase reverse transcriptase; TGF-β, transforming growth factor beta; TOP2A, DNA topoisomerase II alpha.

### Inclusion and exclusion criteria

#### Inclusion criteria

We included clinical and preclinical studies that reported cardiovascular adverse effects or cardiotoxicity mechanisms related to the drug classes of interest. Eligible sources encompassed randomized controlled trials, observational studies, meta-analyses, and systematic reviews evaluating outcomes such as exacerbation of HF, arrhythmias (including QT-prolongation and torsade de pointes), myocardial ischaemia or infarction, and other clinically significant cardiovascular events. We also included pertinent case series or case reports if they illustrated severe or novel cardiovascular reactions. Additionally, mechanistic studies were included if they elucidated pathways of drug-induced cardiovascular adverse effects.

#### Exclusion criteria

We excluded articles that did not specifically address cardiovascular outcomes or mechanisms (e.g. studies focused solely on efficacy or non-cardiac side effects). To maintain the scope of non-oncologic drugs, any studies primarily involving oncologic therapies or cancer patient populations were excluded. Where multiple publications reported duplicative data, the most comprehensive or recent report was included.

### Data synthesis

No formal meta-analysis or quantitative synthesis was performed, given the heterogeneity of drug classes and outcome measures in the retrieved literature. Instead, we adopted a qualitative, narrative synthesis approach.

### Major drug classes implicated

Based on the most commonly used/prescribed drugs^53^ in the last decade, we have selected the following drug groups. For each, we provide the cardiovascular adverse effects, the proposed mechanism, and consideration for practicing physicians. A summary of the most common non-oncologic drugs and their cardiovascular adverse effects can be found in *Table 2*. A more extensive list of drugs that cause cardiovascular adverse effects and proposed mechanisms of action and toxicity are included in *Table 1*. Selected clinically relevant interactions leading to possibly severe cardiovascular toxicity are included in *Table 3*.

**Table 2:** Cardiovascular adverse effects of common non-oncologic drugs

Drug	Class of drug	Cardiac adverse effects						
		QT prolongation/TdP	Hyper-tension	Brady-cardia	Tachy-cardia	AF/ Arrhythmia	LV Dysfunction	Other
Macrolides: azithromycin, clarithromycin, levofloxacin, moxifloxacin	Antimicrobials	✓						
Fluoroquinolones	Antimicrobials	✓						
Chloroquine	Antimalarial	✓						
Hydroxychloroquine	Antimalarial	✓						Conduction abnormalities, cardiomyopathy
Azidothymidine	Antiviral							
Dolutegravir and bictegravir	Antiviral							Gain weight, mild effect on lipids
Ritonavir-boosted lopinavir	Antiviral							Unfavourable lipid changes and increased cardiovascular event risk
Tenofovir alafenamide	Antiviral							More weight gain and less favourable lipid profiles
Abacavir	Antiviral							Increased CV risk
Diclofenac	Traditional Selective COX-2 inhibitors							Increased risk of cardiac arrest
Ibuprofen, naproxen, and indomethacin	Nonselective COX-1/COX-2 inhibitors							Increased CV risk, increased risk of cardiac arrest
Naproxen	Nonselective COX-1/COX-2 inhibitors							Safest alternative
Selective COX-2 inhibitors	Selective COX-2 inhibitors							Increased CV risk
Etanercept, infliximab, adalimumab, certolizumab pegol, and golimumab	TNF alpha (TNFα) inhibitors						✓	
Glucocorticoid	Glucocorticoid		✓				✓	Increased CV risk
Rosiglitazone and pioglitazone	Thiazolidinediones						✓	
Saxagliptin and sitagliptin	DPP-4 inhibitors						✓	
liraglutide	GLP-1 Agonist					✓		In patients with HFrEF, arrhythmia?
Nifedipine	Dihydropyridine calcium channel antagonists						✓	
Amlodipine							✓	
Diltiazem, and verapamil	Non-dihydropyridines			✓			✓	
Propranolol, labetalol, acebutolol	*β*-adrenoceptor blockers			✓			✓	Bradycardia, atrioventricular blockade, hypotension, or HF
Prazosin and doxazosin	α1-blockers					✓		Hypotension
Digoxin	Cardiac glycoside			✓				Mild intoxication, bradycardia and atrioventricular block more severe intoxications, atrial and ventricular tachyarrhythmias
Disopyramide	Class I antiarrhythmics						✓	
Flecainide	Class I antiarrhythmics						✓	
Sotalol, ibutilide, dofetilide, almokalant,	Class III antiarrhythmics	✓						
Amiodarone, and dronedarone	Class III antiarrhythmics	✓						
Flecainide	Class I antiarrhythmics						✓	

AF, atrial fibrillation; CV, cardiovascular; HF, heart failure; HFrEF, heart failure with reduced ejection fraction; LV, left ventricular, Tdp, torsade de pointes.

**Table 3:** Selected clinically relevant interactions leading to possibly severe cardiovascular toxicity

Drug 1	Drug 2	Relevant cardiovascular risk	Mechanism
Amiodarone	Nondihydropyridine Ca2+2+ channel blockers	Sinus arrest	Potentiation of negative chronotropic and inotropic effect
Amiodarone	Some quinolones	Torsade de pointes	Additive effect on QT interval
Amiodarone	Some β-blockers (metoprolol, carvedilol)	Hypotension, bradycardia, asystole, possibly ventricular fibrillation	Additive effect on the heart and inhibition of CYP2D6 by amiodarone
Amiodarone	Sotalol	Torsade de pointes, hypotension	Excessive bradycardia can facilitate torsade de pointes
Drugs prolonging QT	Drugs lowering plasma potassium concentration (amphotericin B, β2-agonists, corticosteroids, loop and thiazide diuretics, theophylline, misuse or overuse of laxatives)	Torsade de pointes	Synergistic effect
β-Blockers	Cholinomimetics	Bradycardia, AV blocks, and hypotension	Synergistic negative chronotropic effect
β-Blockers	Nondihydropyridine Ca2+2+ channel blockers	Bradycardia, asystole, sinus arrest	Additive effect on the heart
β-Blockers	Digoxin	Bradycardia, AV block	Additive effect
β-Blockers	Dronedarone	Bradycardia	Slowed heart rate; dronedarone can inhibit CYP2D6 altering metabolism of some β-blockers
β-Blockers	Antipsychotics-phenothiazines	Hypotension	Additive effect
β-Blockers	Propafenone	Profound hypotension and cardiac arrest	Propafenone can inhibit metabolism of some β-blockers through inhibition of CYP2D6
Some β-blockers	Some SSRI	Bradycardia, AV blocks, hypotension	Fluoxetine and paroxetine are inhibitors of CYP2D6 and thus slow metabolism of some β-blockers
Calcium channel blockers	Azoles, clarithromycin, some HIV-protease inhibitors	Hypotension and/or bradycardia	Inhibition of metabolism of Ca2+2+ channel blockers
Digoxin	Amiodarone	Torsade de pointes also arrhythmia	Torsade de pointes might by facilitated by bradycardia caused by digoxin; amiodarone blocks P-glycoprotein,
Digoxin	Azoles, clarithromycin, some HIV-protease inhibitors	Arrhythmia	Inhibition of P-glycoprotein
Digoxin	Nondihydropyridine Ca2+2+ channel blockers	Bradycardia, asystole, sinus arrest	Inhibition of P-glycoprotein, synergistic effect on the heart
Digoxin	Loop or thiazide diuretics, amphotericin B, corticosteroids	Arrhythmia	Hypokalemia potentiates digoxin toxicity
Digoxin	Ix. Calcium	Arrhythmia	Hypercalcemia increases effect of cardiac glycosides
Digoxin	Propafenone	Arrhythmia	Possible inhibition of P-glycoprotein by propafenone

AV, atrioventricular; CYP2D6, cytochrome P450 2D6; PD, pharmacodynamics; PK, pharmacokinetics; SSRI, selective serotonin reuptake inhibitor.

### Antimicrobials

#### Macrolides and fluoroquinolone^54,55^

Macrolides and fluoroquinolones have long been associated with increased risk of cardiovascular events and death.^56^ Macrolide use has also been associated with QT prolongation, and many case reports describe cardiac arrhythmias.^57,58^ Nonetheless, multiple large retrospective clinical studies investigating the potential risk of the use of macrolides in clinical practice have yielded conflicting results.^28^ A study of 185 010 high-risk Medicare beneficiaries recorded prescriptions for azithromycin, clarithromycin, levofloxacin, moxifloxacin, doxycycline, and amoxicillin-clavulanate, assessing cardiac risk associated with treatment using macrolides, fluoroquinolones, and other antimicrobials. In unadjusted analyses, macrolides and fluoroquinolones were associated with a risk of cardiac events; however, the risk associated with macrolide was substantially attenuated after adjustment for a wide range of variables, and the risk with fluoroquinolones was no longer statistically significant.

A recent meta-analysis that included 13 studies^54^ showed that fluoroquinolone use was associated with a statistically significant 85% increase in the risk for arrhythmia [odds ratio (OR) 1.85; 95% confidence interval (CI) 1.22–2.81] and a 71% risk increase for cardiovascular mortality (OR 1.71; 95% CI 1.39–2.09). Moxifloxacin use was associated with the highest probability for cardiovascular adverse events. Macrolides and fluoroquinolones cause torsade de pointes primarily through inhibition of the rapid component of the delayed rectifier potassium current, resulting in action potential duration prolongation and increasing susceptibility to early afterdepolarizations, which can trigger torsade de pointes via phase 2 re-entry.^59^

### Antimalarials

Antimalarial drugs such as quinine, chloroquine, hydroxychloroquine, artesunate-amodiaquine, and dihydroartemisinic-piperaquine have been associated with prolongation of QT/QTc interval.^60,61^ Chloroquine and hydroxychloroquine inhibit potassium efflux channels at the level of ventricular cardiomyocytes in a dose-dependent manner, potentially leading to QTc prolongation as early as 2 days after starting the medications.^61^ The incidence of QTc prolongation after starting hydroxychloroquine ranges from 3.9% to 8.5%; however, change in the QTc is not associated with increased mortality. Other cardiac toxicities associated with hydroxychloroquine/chloroquine include conduction abnormalities (3.3% of reported adverse events) and cardiomyopathy (3.4% of reported adverse events), which are dose-dependent and usually occur in the setting of prolonged use (mean treatment duration of 7–12 years). Atrioventricular conduction defects are typically permanent. Cardiomyopathy associated with hydroxychloroquine/chloroquine is typically restrictive, and endomyocardial biopsy is often necessary for diagnosis, but it may improve with drug cessation and guideline-directed medical therapy.

### Antivirals

One major concern of antiviral therapy is mitochondrial toxicity in the liver, skeletal muscles, and heart.^62^ For example, azidothymidine, an antiretroviral human immunodeficiency virus treatment, has been shown to induce mitochondrial dysfunction, leading to mitochondrial fragmentation and an impaired fusion–fission cycle.^63^ Azidothymidine inhibits mitochondrial DNA polymerase and also reverse transcriptase.^62^ Sofosbuvir, an inhibitor of RNA polymerase nonstructural protein 5B used to treat hepatitis C, has been recently reported as cardiotoxic in post-marketing surveillance,^32^ although the mechanism remains unknown.

Newer integrase strand transfer inhibitors like dolutegravir and bictegravir have been linked to greater weight gain, particularly when combined with tenofovir alafenamide, though they generally have a neutral to mild effect on lipids and no increased risk of cardiovascular events. Older protease inhibitors, especially ritonavir-boosted lopinavir, are associated with unfavourable lipid changes and increased cardiovascular event risk, while tenofovir alafenamide leads to more weight gain and less favourable lipid profiles compared with tenofovir disoproxil fumarate. Abacavir use is also associated with a higher incidence of major adverse cardiovascular events (MACEs).^64^

### Immunosuppressants and anti-inflammatory drugs

#### Non-steroidal anti-inflammatory drugs

Non-steroidal anti-inflammatory drugs are the most common medications used for pain, and their mechanism of action involves the blockade of prostaglandin synthases (COX-1 and COX-2), either one or both. The discovery of selective COX-2 inhibitors, called coxibs, reduced gastrointestinal side effects, but were later found to be associated with cardiovascular side effects.^65^ In 2004, the United States Food and Drug Administration (FDA) withdrew the COX-2 inhibitor rofecoxib from the market because of an excess risk of MI and stroke.^66^ Although its significant cardiovascular adverse effects seem to be linked to its unique metabolism,^67^ in 2007, the FDA issued a non-approval letter for the selective COX-2 inhibitor etoricoxib, due to cardiotoxic concerns. Long-term administration of both selective COX-2 inhibitors (e.g. diclofenac) and nonselective COX-1/COX-2 inhibitors (e.g. ibuprofen, naproxen, and indomethacin) has been shown to increase cardiac arrest risk.^37^

The ‘traditional’ NSAID diclofenac is as COX-2 selective as celecoxib and increases cardiovascular risk dose dependently. Randomized trials comparing COX-2 inhibitors with NSAIDs have exaggerated their gastrointestinal benefits by using maximal NSAID doses regardless of indication and/or hidden the cardiovascular risk by comparing with COX-2 selective diclofenac instead of low-dose ibuprofen or naproxen.^68^ Observational studies showed increased cardiovascular risks within weeks of treatment with COX-2 inhibitors and high doses of NSAIDs other than naproxen, which is the safest alternative. COX inhibitors should be used intermittently at the lowest effective dosage,^69^ especially among individuals with increased cardiovascular risk.^68^

Gibson *et al*.^70^ conducted an analysis of the ExTRACT–TIMI 25 trial, which demonstrated that among ST-segment elevation MI patients treated with a fibrinolytic agent and aspirin, use of NSAIDs in the week preceding the incident event was associated with a higher occurrence of recurrent MI; the composite of death and MI; and the composite of death, MI, severe HF, and shock at 30 days.

Two mechanisms have been associated with the cardiovascular side effects of these drugs: (i) an imbalance between the production of pro-aggregatory thromboxane A2 formed by platelets via COX-1 and antiaggregatory active prostacyclin formed via endothelial cyclooxygenases with an involvement of COX-2^71,72^ and (ii) blockade of the synthesis and consequent lack of prostaglandin E2 effect in the kidney. Involvement of COX-2 in the cardiovascular effects is clear, since this pathway is potentially blocked by all known NSAIDs with the exception of low doses of acetylsalicylic acid (aspirin).^73^ Therefore, all NSAIDs can increase blood pressure and may also cause or worsen HF.^74–76^ On the other hand, the imbalance between the thromboxane A2 and the prostacyclin system is influenced differently based on the degree of inhibition of COX-1 and −2, duration of action, and reversibility of inhibition. Regarding NSAIDs, only acetylsalicylic acid in low doses is used in primary prevention of cardiovascular disease^77^ because it irreversibly blocks platelet COX-1 without having a significant effect on endothelial COX-2.^73^ Conversely, coxibs (which are selective for the COX-2) decrease prostacyclin production, without having an important effect on COX-1-mediated thromboxane A2 production.

#### Tumour necrosis factor inhibitors

Since their approval for treatment of rheumatoid arthritis, tumour necrosis factor (TNF) alpha (TNFα) inhibitors have been associated with cardiovascular adverse effects.^78,79^ A search of the FDA Adverse Event Reporting System for HF associated with using etanercept, infliximab, adalimumab, certolizumab pegol, and golimumab in the past 10 years^80^ yielded 594 898 records of adverse events related to anti-TNF medication; of these, 2869 were HF. More than half of these events were in women (mean age of 64). Adalimumab was the most used drug (73.9%) among those who developed HF. Etanercept [reported odds ratio (ROR) = 4.6; 95% CI 4.0–5.3] and adalimumab (ROR = 1.3; 95% CI 1.2–1.3) were significantly associated with HF. Additionally, a systematic review^81^ of patients with rheumatoid arthritis and HF that included observational studies and a meta-analysis, found no increase in the risk of incident or worsening HF in patients treated with tumour necrosis factor-α inhibitors (infliximab, etanercept, and adalimumab) except for patients ≥65 years, who had a higher risk of HF hospitalization (HR 1.7; 95% CI 1.07–2.69) and death (HR 4.19; 95% CI 1.48–11.89).^82,83^

The effects of TNF-α in the heart are concentration dependent via two pathways, and depend on the receptor to which it binds: TNFα-1 (TNFR1), which is an apoptotic receptor, or TNFα-2 (TNFR2), which is a cardioprotective receptor.^84^ A study found that while inhibition of TNFR1 is cardioprotective, the greater inhibition of TNFR2 may be the underlying mechanism of the cardiovascular risks associated with TNF-α inhibitors.^85^

#### Corticosteroids

Exogenous administration of glucocorticoids is associated with hypertension in about 20% of cases.^86^ Additionally, there is conflicting evidence about the use of glucocorticoids and the risk of MACE. A recent systematic review and meta-analysis^87^ that assessed the association between glucocorticoids and cardiovascular risk, including MACE, provided two major findings. First, that glucocorticoids were associated with increased risks of MACE, coronary heart disease, and HF. Second, the risk of MACE increased with increasing cumulative or daily doses of glucocorticoids.

### Antidiabetic medications

#### Thiazolidinediones

Thiazolidinediones (e.g. rosiglitazone and pioglitazone) are proliferator-activator receptor gamma agonists that modulate the transcription of insulin-sensitive genes involved in the control of glucose and lipid metabolism. In an analysis of published literature, the FDA website, and a clinical trials registry maintained by the drug manufacturer of rosiglitazone published in 2007,^41^ rosiglitazone was associated with a significantly increased risk of MI and a borderline increased risk of death from cardiovascular causes. Recent meta-analyses confirmed that thiazolidinediones worsen existing HF and increase the risk for new-onset HF.^88–94^ Similarly, in a retrospective analysis of 227 571 Medicare beneficiaries treated with a thiazolidinedione,^95^ authors found that the risk of HF was greater with rosiglitazone compared with pioglitazone (HR 1.25; 95% CI 1.16–1.34).

Mitochondrial dysfunction has been associated with the unfavourable effects of thiazolidinedione.^96^ There are new insights into the mechanism of cardiovascular adverse effects using human-derived cardiomyocytes. The authors found a significant loss of mitochondrial adenosine triphosphate production upon exposure to either pioglitazone and rosiglitazone in the cytotoxicity assay. Their toxicoproteomics analysis revealed that mitochondrial dysfunction primarily stems from oxidative phosphorylation impairment, with distinct signalling mechanisms observed for both agents. The type of cell death differed strikingly between the two agents, as well as additional mechanistic aspects of cardiovascular adverse effects, showcasing drug specificity.

#### Dipeptidyl peptidase IV inhibitors

The use of Dipeptidyl peptidase IV (DPP-4) inhibitors (e.g. saxagliptin and sitagliptin) in patients with type 2 diabetes mellitus has been associated with increased risk of HF, which seems to be a class effect of these drugs, although the severity of risk may be influenced by concomitant therapy.^97,98^ Several mechanisms could contribute to this deleterious effect, including activation of the sympathetic nervous system to stimulate β-receptor signalling and cause cardiomyocyte cell death. Inhibition of DPP-4 potentiates the actions of SDF-1, NPY, and substance P to increase sympathetic nerve traffic and (through β-receptor stimulation) could cause cardiomyocyte injury and loss (through a CaMKII mechanism).

#### GLP-1 agonist

Recently, controversial signals from *post hoc* analyses in trials using GLP-1 agonists has been published, due to positive chronotropic effect causing an increase in heart rate. A *post hoc* analysis of the FIGHT trial suggested that in patients with HF with reduced ejection fraction (HFrEF), liraglutide might increase the risk of cardiovascular adverse effects, possibly driven by excess risk of arrhythmias and worsening HF events.^99^ Similar results from were seen in the LIVE Study, where liraglutide failed to show any improvement in ejection fraction (EF) after 24 weeks of exposure to liraglutide (primary objective). Furthermore, there was no change in left ventricular (LV) dimensions, New York Heart Association (NYHA) class, or quality of life. Moreover, there was a non-significant trend for LV dilation in liraglutide-treated patients.^100^ One possible explanation for this lack of benefit was an increase in heart rate. However, in the EXSCEL trial that evaluated once-weekly exenatide in patients with type 2 diabetes with varying cardiovascular risk (some of whom had HFrEF at baseline), the overall trial was neutral for the MACE composite, and a post-hoc subgroup analysis differentiated the effect based on HF status. In patients without baseline HF, once-weekly exenatide reduced the risk of all-cause mortality, but no such benefit was seen in patients with pre-existing HF. These controversial signals might be related to agent and phenotype-specific effects, thereby precluding class generalization.

### Cardiovascular medications

#### Calcium channel blockers^101^

Dihydropyridine calcium channel antagonists such as nifedipine have both negative inotropic and vasodilating effects because they block the transmembrane influx of calcium ions into cardiac and vascular smooth muscles.^82,83^ In a small trial that assessed the potential benefits of nifedipine in patients with HF, authors found a marked worsening of HF; 5 of 21 patients treated with nifedipine required hospitalization compared with 0 of 20 who received isosorbide dinitrate.^102^ The possible benefit of amlodipine in a subgroup of patients with nonischaemic cardiomyopathy and HF seen in one study was not reproduced in a second study.^103,104^ Nevertheless, in both trials, there were high rates of pulmonary oedema and peripheral oedema in patients treated with amlodipine. Non-dihydropyridines, diltiazem, and verapamil also have negative inotropic effects and can worsen HF more than the dihydropyridine calcium channel blockers because the negative inotropic effects are not offset by vasodilation.^82,83^ In a study of 2466 patients with recent MI randomized to diltiazem or placebo, diltiazem increased the risk of adverse cardiac events (HR 1.41; 95% CI 1.01–1.96) in the subgroup of 490 patients with baseline pulmonary congestion,^105^ which was directly related to the severity of baseline HF; however, caution is necessary in patients with preexisting systolic dysfunction. Diltiazem and verapamil have also been associated with bradyarrhythmia, due to inhibition of the automaticity of the sinus node^59^ and, therefore, should be avoided in patients with preexisting dysfunction in the absence of a functioning pacemaker. Short-acting dihydropyridines, like nifedipine, can produce reflex tachycardia as a result of their rapid and dominant peripheral vasodilator effect.

#### Beta-blockers

*β*-adrenoceptor blockers (antagonists) are considered to be quite safe in recommended doses mainly because of their large therapeutic indices. The introduction of beta-blockers may cause transient worsening of HF symptoms, due to their negative inotropic action. Therefore, the initiation of treatment is recommended after stabilization of HF symptoms. There is also an increased risk of cardiovascular adverse effects, due to interactions with other drugs as shown in *Table 3*,^86^ which might include bradycardia, atrioventricular blockade, hypotension, or HF. Some beta-blockers also have membrane-stabilizing effects (e.g. propranolol, labetalol, acebutolol), and overdose might be associated with more pronounced cardiac toxicity.^106^ In addition to the above-mentioned cardiac effects and lack of consensus about the precise definition, the rebound phenomenon^107^ should be noted, which has been associated with rapid discontinuation of chronically used beta-blockers. The abrupt discontinuation of a beta-blockers is associated with a fourfold increased risk of acute coronary events in hypertensive patients, increased risk of in-hospital death in HF patients, precipitation of angina, and increased risk of death and rehospitalization in patients post-MI.^107^

#### α1-blockers

The α-blockers inhibit postsynaptic α1-adrenergic receptors and relax vascular smooth muscle, resulting in vasodilation, and were initially used as antihypertensive medications.^86^ Currently, agents such as prazosin and doxazosin are primarily used for benign prostatic hypertrophy based on the negative results of clinical trials.^108–110^ In ALLHAT,^108^ the risk of HF doubled (risk ratio 2.04; 95% CI 1.79–2.32; *P* < 0.001) in patients receiving doxazosin compared with those on chlorthalidone, perhaps due to misdiagnosis of vasodilator-induced oedema, a smaller blood pressure reduction with doxazosin, or the unmasking of HF by discontinuing other antihypertensive drugs that were protective against HF.^109^ Likewise, in the Veterans Affairs Vasodilator-HF Trial I (V-HeFT I), hydralazine combined with isosorbide dinitrate decreased mortality and improved left ventricular ejection fraction (LVEF) compared with placebo, whereas prazosin was not associated with any cardiovascular benefits.^110^

#### Digoxin

Digoxin is a cardiac glycoside with a narrow therapeutic index that exerts its primary mechanism of action by inhibiting the sodium-potassium adenosine triphosphatase (Na+/K + ATPase) enzyme, particularly within the myocardium.^111^ This drug is used in HF and as a rate-control agent for the rapid ventricular supraventricular arrythmias. Digoxin is associated with very different types of arrhythmias.^86^ In mild intoxication, bradycardia and atrioventricular block are most commonly observed, due to excessive stimulation of vagal tone. In more severe intoxications, atrial and ventricular tachyarrhythmias could appear, most likely due to delayed afterdepolarization leading to increased automaticity and/or ectopic activity. Digoxin toxicity may originate or be exacerbated by drug interactions (*Table 3*).

### Antiarrhythmic medications

#### Class I antiarrhythmics

Some of the class I antiarrhythmics, sodium channel blockers, are known to be potentially harmful in certain patients. Disopyramide has a negative inotropic effect in patients with HF, causing or worsening the condition.^112^ In addition, one study demonstrated that in 100 patients treated with oral disopyramide for ventricular arrhythmias, 16 (12 with a previous history of HF) developed HF within the first 48 h of therapy.^113^ Flecainide may also depress LV function significantly in patients with impaired LV function. The increased mortality associated with flecainide in CAST suggests that this drug should be avoided in patients with HF or structural heart disease.^44,114–116^

#### Class III antiarrhythmics

Class III antiarrhythmics drugs (e.g. sotalol, ibutilide, dofetilide, almokalant, amiodarone, and dronedarone) slow down delayed repolarization and block human ether-à-go-go-related gene (hERG) channels. Logically, all the drugs in this group prolong the QT interval; consequently, the risk of torsade de pointes is expected and clinically well documented.^117^ Nevertheless, amiodarone and dronedarone only rarely cause torsade de pointes, which could be explained by their ability to also block calcium ion channels and possibly sodium ion channels.^86,118^ In addition, amiodarone may result in atrial fibrillation as a result of its ability to induce thyrotoxicosis^119^ in some patients.

### Mechanisms of injury

Cardiovascular adverse effects are complex because different pathophysiological processes are often involved and overlap.

### Subcellular effects

While the mechanisms of direct myocardial injury caused by anticancer agents have been widely studied, the mechanistic links between non-oncologic treatments and myocardial toxicity remain ill-defined, and evidence is limited. Toxic cardiomyopathy is typically multifactorial, involving disruption of cellular bioenergetics, abnormal calcium handling, generation of reactive oxygen species, and activation of cell death pathways such as apoptosis or necrosis.^120^

#### Disrupted mitochondrial function

Drug-induced mitochondrial dysfunction reduces adenosine triphosphate production, impairs structural integrity, and can trigger cell death through necrosis or apoptosis.^121^ During oxidative phosphorylation, mitochondria also produce reactive oxygen species. In healthy cells, reactive oxygen species act as signalling molecules; however, excessive production due to drug injury, inflammation, or aging, overwhelms antioxidant defenses, leading to oxidative stress and damage to proteins, lipids, and DNA.^121^ Ultimately, the drugs that damage mitochondria do so by inhibiting respiratory complexes of the electron chain (e.g. antipsychotics, local anesthetics, antidiabetics); inhibiting or uncoupling oxidative phosphorylation (e.g. oligomycin); inducing mitochondrial oxidative stress (e.g. fibrates); or inhibiting DNA replication, transcription, or translation (antivirals).^122^

Drugs such as NSAIDs (e.g. diclofenac) can also induce mitochondrial permeability transition pore opening, which collapses the mitochondrial membrane potential, causes swelling, and leads to rupture of the outer mitochondrial membrane, releasing pro-apoptotic proteins into the cytosol and ultimately causing cell death.^123,124^

#### Abnormal intracellular calcium modulation

During excitation–contraction coupling, calcium influx into myofilaments initiates contraction, and calcium removal allows relaxation. In general, positive inotropic agents increase intracellular calcium, and negative inotropic agents decrease intracellular calcium, during systole.^123^ An imbalance in intracellular modulation of calcium caused by potentially toxic substances (e.g. flecainide, non-dihydropyridinic calcium channel blockers^82^) may impair both inotropy (contractile strength) and lusitropy (relaxation), contributing to contractile dysfunction.^125^

#### Oxidative stress

Oxidative stress occurs when reactive oxygen species production exceeds the capacity of antioxidant defenses—both enzymatic (e.g. superoxide dismutase, catalase, glutathione peroxidase) and non-enzymatic (e.g. glutathione, coenzyme Q10, lipoic acid). Persistent oxidative stress damages cellular macromolecules, promotes mitochondrial dysfunction, and accelerates cell death pathways.^126^

#### Apoptosis and necrosis

Cardiomyocyte loss can occur via apoptosis, necrosis, or autophagy, often in overlapping ways. Mitochondria are central to both intrinsic apoptotic signalling through cytochrome c release, and necrotic injury through mitochondrial permeability transition pore opening. Additional cell death modalities, such as ferroptosis (iron-dependent) and pyroptosis (inflammatory), may also be triggered depending on the drug and injury type.^127^

### Electrophysiology

#### Ion channel blockade: hERG potassium channel and QT prolongation

One of the most frequent adverse effects encountered in drug development is drug-induced arrhythmia, due to off-target blockade of voltage-gated cardiac ion channels, particularly the hERG potassium channel (Kv11.1).^10^ The hERG blockade reduces the rapid delayed rectifier potassium current, prolonging phase 3 repolarization of the cardiac action potential and leading to long QT syndrome. Multiple drugs affect the rapid delayed rectifier potassium current, including antibiotics (fluoroquinolones, macrolides), antipsychotics (phenothiazines, butyrophenones), and antidepressants (tricyclic antidepressants, selective serotonin reuptake inhibitors).^128^ This pro-arrhythmic risk makes hERG inhibition a critical ‘anti-target’ in early drug discovery.^129^

### Inflammatory myocarditis: immune checkpoint inhibitors and non-oncologic analogs

Immune checkpoint inhibitors, which are widely used in oncology, enhance immune activity by blocking inhibitory pathways such as PD-1 (e.g. nivolumab)/PD-L1 (e.g. atezolizumab) and CTLA-4 (e.g. ipilimumab). Similar immune-modulating strategies are being explored for non-cancer indications; however, immune checkpoint inhibitors can provoke immune-related adverse events, including myocarditis, through excessive immune activation against cardiac tissue.^130^ Although most cases respond to corticosteroids, fulminant myocarditis can cause severe morbidity or death.^131^

### Haemodynamic stress: fluid retention and hypertension

Numerous drugs can cause peripheral oedema through mechanisms such as increased capillary hydrostatic pressure, sodium/water retention, lymphatic insufficiency, and increased capillary permeability (treprostinil, ciclosporin, tacrolimus).^132^ The potential pathophysiological mechanisms by which different drugs result in peripheral oedema can be heterogeneous depending on the drug. For example, sodium water retention can be due to mineralocorticoid effect (e.g. abiraterone), intrinsic anti-natriuretic effect, renin–angiotensin–aldosterone system activation (e.g. insulin), and in some cases, is not fully understood.^132^ Certain medications also induce secondary hypertension, often overlooked in clinical practice. Mechanisms include renin–angiotensin–aldosterone system activation (e.g. oral contraceptive pills with oestrogen component), sympathetic overactivity (e.g. methylphenidate), and alteration in peripheral vascular tone (e.g. NSAIDs, glucocorticoids).^133^

### Autonomic imbalance: sympathomimetic effects

Sympathomimetic drugs mimic adrenergic stimulation, increasing heart rate, contractility, and vascular tone via β1- and α1-adrenoceptor activation. Some agents such as dextroamphetamine and lisdexamfetamine also promote norepinephrine release, further amplifying cardiovascular effects. These changes markedly elevate myocardial oxygen demand, potentially precipitating ischaemia or arrhythmias in susceptible patients.^86^

### Fibrosis

An association between certain drugs and valvular fibrosis has been described. These drugs include appetite suppressants (e.g. fenfluramine and dexfenfluramine), dopamine agonists (e.g. pergolide and cabergoline), and the recreational drug ecstasy (methylenedioxymethamphetamine).^121^ A likely mechanism seems to be interference with serotonin metabolism, as well as its associated receptors and transporter gene, leading to cell proliferation and subsequent valvular heart disease.^134^

### Practical considerations

**Figure 1:**
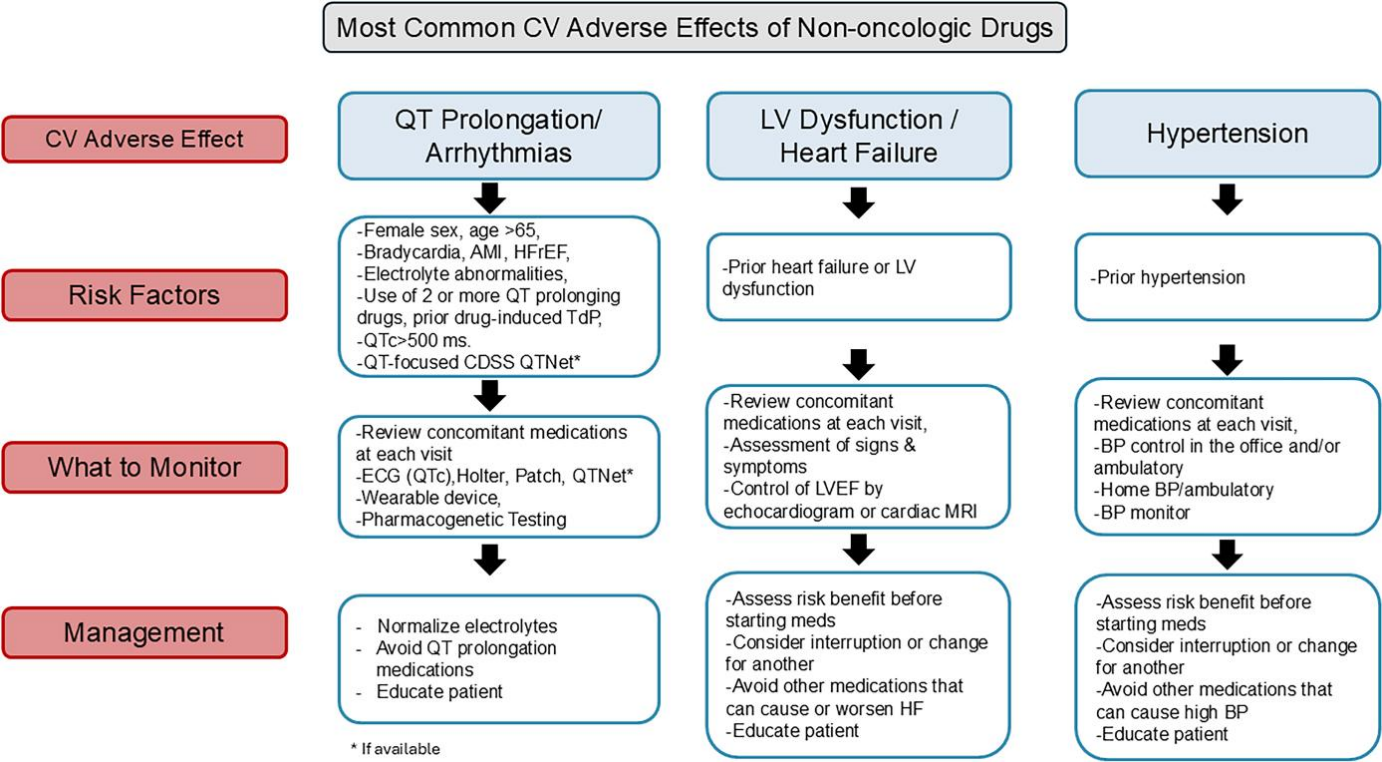
**Mechanisms of underlying cardiovascular adverse effects associated with non-oncologic medications.** This figure displays the mechanisms of cardiotoxicity associated with non-oncologic medications including direct myocardial damage, haemodynamic stress, electrophysiology, fibrosis, inflammatory, and autonomic imbalance. AMI, acute myocardial infarction; BP, blood pressure; CDSS, clinical decision support systems; ECG, electrocardiogram; HF, heart failure; HFrEF, heart failure with reduced ejection fraction; LVEF, left ventricular ejection fraction; MRI, magnetic resonance imaging; QT-focused CDSS, QT interval–focused Clinical Decision Support Systems; QTNet, QT Interval Network; Tdp, torsade de pointes.

*Figure 1* provides a simple guide that outlines steps to minimize cardiovascular adverse effects of common non-oncologic drugs. Physicians should assess the patient’s baseline cardiovascular risk including a detailed history and physical exam, concomitant medications, and tests [e.g. electrocardiogram (ECG) for QT interval, serum electrolytes, and renal function]. Depending on the prescribed drug, there should be a monitoring plan in place that might include serial ECG, blood pressure control, or LVEF assessment,^59,82^ with concomitant medications always being reviewed. For example, when prescribing agents known to confer torsadogenic risk, including macrolides, fluoroquinolones, and certain antipsychotics, baseline QTc and electrolyte assessment should be performed, and combinations with other QT-prolonging drugs should be avoided whenever possible. For those agents that increase haemodynamic load or increase HF risk (such as NSAIDs/COX-2 inhibitors, DPP-4 inhibitors, thiazolidinediones, certain calcium channel blockers), careful baseline evaluation and close longitudinal monitoring of HF status are warranted, with attention to body weight, blood pressure, and clinical signs or symptoms of congestion.

Management of the cardiovascular adverse effect will depend on the type of condition, as well as reassessing the risk benefit of the drug indication, considering interruption, change, or specific treatment. Specific resources, such as the European Heart Rhythm Association Practical Compendium of Antiarrhythmic Drugs offers detailed advice on the usage, monitoring, and management of these medications in clinical practice.^135^

### Pharmacogenomic testing, artificial intelligence–driven pharmacovigilance, and QT prolongation prediction in cardiovascular pharmacotherapy

Combining pharmacogenomic testing with real-world drug safety monitoring is changing how cardiovascular side effects are predicted and prevented. Genetic testing before treatment can help identify patients more likely to experience cardiovascular adverse effects. This approach supports safer, more individualized therapy. There is evidence indicating that implementation of pharmacogenomic testing in clinical practice reduces serious adverse reactions and improves overall treatment safety.^136^ Updated international guidelines also promote the integration of genetic data into electronic health records and national pharmacovigilance programs.^137^ For example, CYP2D6 metabolizer status can inform tricyclic antidepressant dosing by identifying patients at increased risk of drug accumulation and QRS prolongation.^138^

Additionally, AI is further transforming this field by enhancing detection and prediction of cardiovascular risks. QT-focused clinical decision support systems (CDSS) embedded within electronic health records can aid in the prevention of drug-induced long QT syndrome by identifying high-risk patients at the time of prescribing and prompting appropriate ECG monitoring and mitigation strategies.^139^ Deep-learning systems such as QTNet can analyse routine ECGs to accurately predict which patients may develop prolonged QT intervals after starting QT-prolonging medications, achieving strong predictive performance within days of exposure.^140,141^

Within the workflow in *Figure 1*, QT-focused CDSS and QTNet-type models can be embedded to identify high-risk QT prolongation scenarios, prompt appropriate baseline assessment and monitoring, and individualize follow-up while complementing clinical judgment.

### Future directions

Using AI and genetic testing together provides new ways to prevent cardiovascular adverse effects caused by commonly used/prescribed medications. Integrating AI tools with pharmacogenomic information might offer a powerful strategy for early identification of at-risk patients and prevention of life-threatening arrhythmias. Together, these advances are shifting cardiovascular pharmacotherapy towards a more proactive and personalized approach to drug safety. Studies are needed to confirm how these methods improve patient outcomes, help clinicians select the right drugs, and reduce hospitalizations linked to drug-induced cardiovascular events. As these technologies become more widely used and tested, cardiovascular pharmacotherapy will increasingly shift from reporting adverse effects to preventing them altogether.

## Conclusion

Non-oncologic medications constitute a key pillar of modern therapeutics; yet, their potential for cardiovascular adverse effects represents a clinically relevant and often underrecognized challenge. This review highlights that such adverse effects are not confined to oncologic agents, but extends to commonly used drugs such as antibiotics, central nervous system therapies, antidiabetic agents, anti-inflammatory drugs, and other widely prescribed treatments, including cardiovascular drugs themselves. Looking ahead, advances in systems pharmacology, pharmacogenomics, and digital health tools hold the potential to transform the way we identify and manage drug-induced cardiovascular risk. Integration of electronic health record-based surveillance, AI-driven signal detection, and patient-specific risk stratification could allow earlier recognition of cardiovascular adverse effects and safer therapeutic decision-making. Collaborative efforts between cardiologists, pharmacologists, and regulatory agencies will be essential to translate these innovations into clinical benefit and to ensure that the expanding arsenal of non-oncologic medications is deployed without compromising cardiovascular safety.

## Data Availability

This review is based on previously published studies. All tables and figures were derived from data reported in the cited literature. No new datasets were generated or analysed.
